# Vitamin D profile in healthy children aged 7-12 years old in Indonesia

**DOI:** 10.1186/1687-9856-2013-S1-P167

**Published:** 2013-10-03

**Authors:** Frida Soesanti, Aman Pulungan, Bambang Tridjaja, Jose RL Batubara

**Affiliations:** 1 Pediatric Endocrinology Division, Faculty of Medicine, Universitas Indonesia, Ciptomangunkusumo Hospital, Jakarta, Indonesia

## Background

The recent data on the vitamin Dshowed a surprising result, which exhibited in high prevalence of vitamin D deficiency and insufficiency in children and adolescence. Thisnot only occurs in country that lies in high latitude but also in sun rich country. Many factors contributing to this condition, including changing in life style. No data available regarding vitamin D status in healthy children in Indonesiaand this will be the first study addressing this issue.

## Aims

To find out vitamin D profile of healthy children in Indonesia andfactors associated with vitamin D status in those children.

## Methods

This was a cross sectional study involving 120 children aged 7-12 years from two different elementary schools in Jakarta. We used structured questionnaire on life style and performa thorough clinical examinations to all participants. We measured the serum level of calcium, phosphate, bone-alkaline phosphatase (BALP), and 25(OH)D. The 25(OH)D level classified as sufficient if ≥ 32 ng/dL, insufficient 15-31 ng/dL, and deficient if <15 ng/dL. Sun-exposurewas analyzed based on duration of exposure per week.The association between vitamin D and contributing factors was analyze using chi-square with significant value at 0.05.

## Results

Of 120 children (45 boys and 75 girls), 78.3% were classified as brown (4^th^ degree of Fitzpatrick scale) in skin tone. Most of the children (52.5%) were well-nourished, while 21.7% were obese.Vitamin D insufficiency was found in **75.8%** of the subjects, while 15% of the subjects were classified as vitamin D deficiency. Calcium level was low in16.7% subjects, while the phosphate level was found to be in the normal range for all subjects. Bone alkaline phosphatasewas normal in 31.6% subjects. Vitamin D status was not associated with duration of sun exposure (*x^2^*, P=0.143), and there were no difference in vitamin D status between the obese and non-obese subjects (P=0,65). Skin tone, clothing style, sunblock usage, and milk consumption did not influence vitamin D status (P=0.08, P=0.43, P=0.05, respectively).

## Conclusion

There is high prevalence of vitamin D insufficiency in healthy children aged 7-12 years in Indonesia. Girls have increased risk compare to boys. This result should increase the awarenessof health professional and society regarding vitamin D status in Indonesian children.

